# Cryogenic superresolution correlative light and electron microscopy of vitreous sections

**DOI:** 10.52601/bpr.2022.220005

**Published:** 2022-08-31

**Authors:** Buyun Tian, Maoge Zhou, Fengping Feng, Xiaojun Xu, Pei Wang, Huiqin Luan, Wei Ji, Yanhong Xue, Tao Xu

**Affiliations:** 1 National Laboratory of Biomacromolecules, CAS Center for Excellence in Biomacromolecules, Institute of Biophysics, Chinese Academy of Sciences, Beijing 100101, China; 2 University of Chinese Academy of Sciences, Beijing 100049, China; 3 National Research Center for Rehabilitation Technical Aids, Beijing 100176, China

**Keywords:** Cryogenic, Superresolution, Fluorescence microscopy, Electron microscopy, Correlative light and electron microscopy (CLEM), Electron tomography

## Abstract

Fluorescence microscopy and electron microscopy complement each other as the former provides labelling and localisation of specific molecules and target structures while the latter possesses excellent revolving power of fine structure in context. These two techniques can combine as correlative light and electron microscopy (CLEM) to reveal the organisation of materials within the cell. Frozen hydrated sections allow microscopic observations of cellular components *in situ* in a near-native state and are compatible with superresolution fluorescence microscopy and electron tomography if sufficient hardware and software support is available and a well-designed protocol is followed. The development of superresolution fluorescence microscopy greatly increases the precision of fluorescence annotation of electron tomograms. Here, we provide detailed instructions on how to perform cryogenic superresolution CLEM on vitreous sections. From fluorescence-labelled cells to high pressure freezing, cryo-ultramicrotomy, cryogenic single-molecule localisation microscopy, cryogenic electron tomography and image registration, electron tomograms with features of interest highlighted by superresolution fluorescence signals are expected to be obtained.

## INTRODUCTION

“It is very easy to answer many of these fundamental biological questions; you just look at the thing!” said Richard Feynman (1960), the renowned physicist half a century ago. If we hope to gain knowledge about the mechanisms underlying cellular functions, we must understand what components are in the cell, where they are located, what their structures are, and how they are organised. Direct observations of the interior of cells with microscopic imaging techniques have provided the most convincing evidence on these issues.

Electron microscopy (EM) reveals fine structures of subcellular components and biomacromolecules at nanometre or angstrom resolution. Fluorescence microscopy (FM), although not as powerful as EM in spatial resolution, performs excellently at localising specific molecules and labelling subcellular structures of interest. This advantage makes FM a useful complement to EM, since the identification of target structures on greyscale electron micrographs is challenging in many cases, while fluorescence micrographs may serve as a guide. It is beneficial to apply both techniques to the same specimen so that FM can (1) navigate the acquisition of electron micrographs and (2) create image overlays on electron micrographs that annotate specific structures, while EM reveals the ultrastructure in detail. This concept is realised with correlative light and electron microscopy (CLEM) (Briegel *et al*. 2010; de Boer *et al.* 2015; Scher and Avinoam 2021).

A major concern in CLEM is the compatibility of the sample preparation methods. Conventional EM sample preparation usually involves chemical fixation, heavy metal staining, dehydration, resin embedding, *etc*. These harsh treatments have a detrimental effect on fluorescence preservation. To optimise sample preparation procedures or use EM-compatible fluorescent proteins may keep the balance between the preservation of the fluorescence and the ultrastructure (de Boer *et al*. 2015; Fu *et al*. 2020; Kukulski *et al*. 2012; Paez-Segala *et al*. 2015; Watanabe *et al*. 2011). Cryofixation, on the other hand, fixes the sample in a vitrified hydrated state that is fully compatible with FM and EM imaging (Briegel *et al*. 2010; Li *et al*. 2018; Wang *et al*. 2017). Because the sample is not subjected to harsh treatments and preserves the aqueous environment, which is vital to the maintenance of a natural cellular structure, it can be observed and studied close to a native state. Under cryogenic conditions, photobleaching, which is often undesirable in FM, is reduced (Schwartz *et al*. 2007). Cryogenic samples are also suitable for electron tomography (ET), a specialised application of transmission electron microscopy that reveals subcellular ultrastructure in 3D (Asano *et al*. 2015; Orlova and Saibil 2011). The cryogenic temperature reduces radiation damage induced by the electron beam and thus increases the effective resolution of the image (Lucic *et al*. 2005; Milne *et al*. 2013). With the aforementioned advantages, cryo-CLEM is believed to be a practical and effective realisation of CLEM.

Another concern is the resolving power of light microscopy. Due to the diffraction of light, the best possible lateral resolution of conventional light microscopy is approximately 200 nm, which is not comparable to the resolution of tomograms produced by cryo-ET. This fact indicates that although conventional cryo-FM may serve as a guide for cryo-ET data acquisition, the images it produces are usually too rough to be superimposed on cryo-ET images. Fortunately, the photoswitching or photoblinking properties of many fluorophores are retained at cryogenic temperatures (Hoffman *et al*. 2020; Liu *et al*. 2015; Tuijtel *et al*. 2019), enabling the performance of single-molecule localisation microscopy (SMLM), a superresolution technique whose resolution is an order of magnitude better than that of conventional FM (Betzig *et al*. 2006; Hess *et al*. 2006; Rust *et al*. 2006). SMLM can be integrated into the cryo-CLEM scheme so that superresolution fluorescence micrographs can precisely annotate electron tomograms (Chang *et al*. 2014; Dahlberg and Moerner 2021; Dahlberg *et al*. 2020; DeRosier 2021; Liu *et al*. 2015; Tian *et al*. 2021; Tuijtel *et al*. 2019).

SMLM-based cryogenic superresolution CLEM (csCLEM) is a useful technique for revealing subcellular ultrastructure and the distribution of macromolecules *in situ*. This integrated technique combines the specificity and precision of SMLM and the high resolution of cryo-ET to study cells and cellular components in a near-native state. Its possible applications include revealing how specific molecules are organised in their cellular context to perform their functions, identifying and visualising subcellular structures of interest, pinpointing target macromolecules in tomograms for *in situ* structural analysis, *etc*. Several pioneering studies have demonstrated the practicability of csCLEM through delicate design and professional practice (Chang *et al*. 2014; Dahlberg *et al*. 2020; Liu *et al*. 2015; Tuijtel *et al*. 2019), but the technical complexity has hampered its widespread application by cell and structural biologists. Here we present a protocol for csCLEM based on our homemade ultrastable cryogenic superresolution fluorescence imaging system (Xu 2019; Xu *et al*. 2018). We hope that the practical workflow developed by ourselves will be helpful for targeted subcellular imaging and structural analysis *in situ*. U2OS cells with endoplasmic reticulum (ER)–mitochondrial membrane contact sites highlighted by green fluorescence (Yang *et al*. 2018) are used as an example of suitable biological samples, but this protocol is expected to apply to other samples with fluorescence labels.

## OVERVIEW OF THIS PROTOCOL

**Figure 1:**
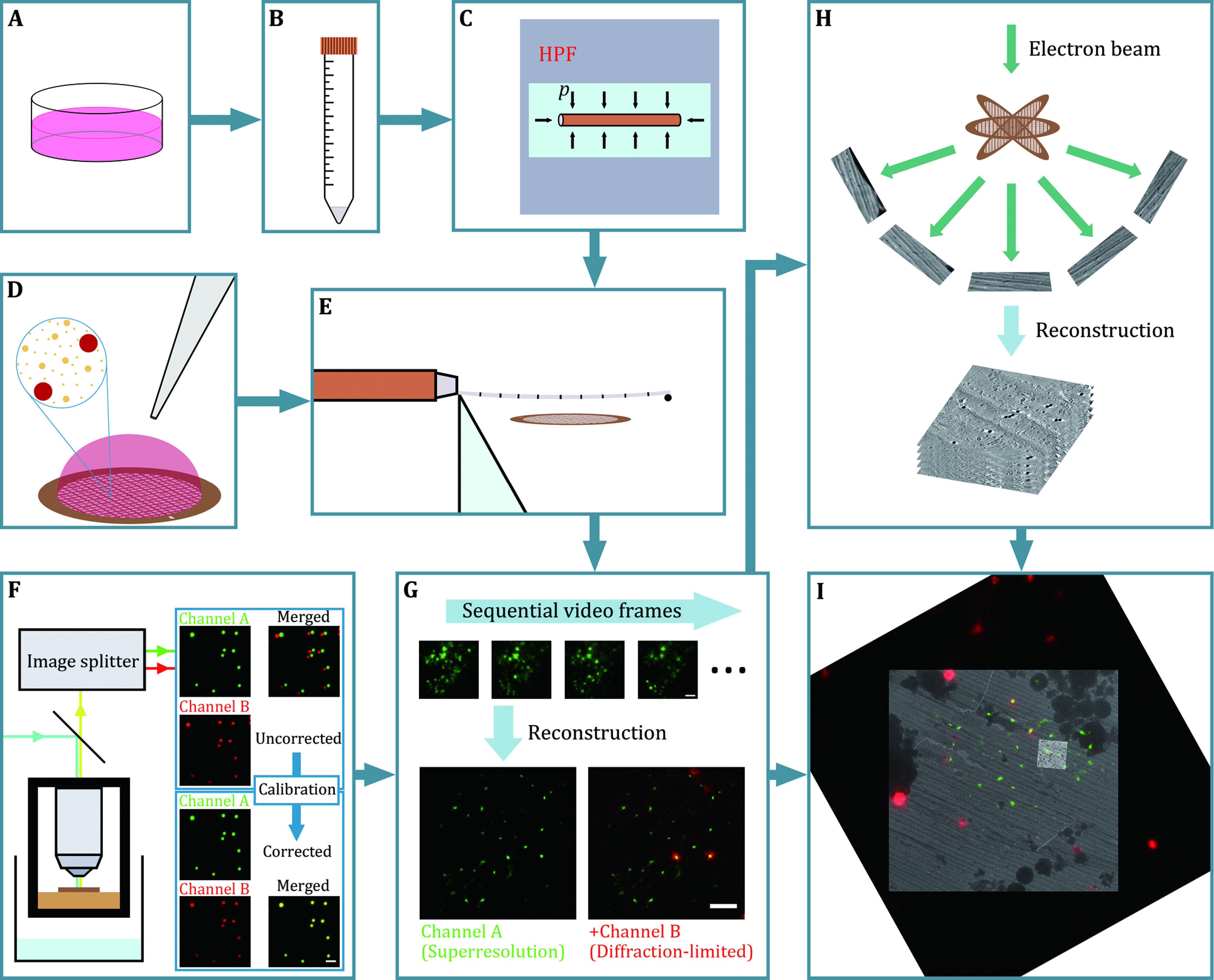
Overview of the protocol. **A**–**E** Sample preparation. Fluorescence-labelled cells (**A**) are centrifuged, resuspended in dextran solution (**B**) and vitrified with a high-pressure freezer (**C**). **D** 0.2 μm dark red fluorescent microspheres (red spheres in the inset), 50 nm gold nanoparticles (larger golden dots in the inset) and 10 nm gold nanoparticles (smaller golden dots in the inset) are applied over the EM grid as fiducial markers for image registration and tomographic tilt series alignment. **E** The vitrified sample is sectioned with a cryo-ultramicrotome, and the cryosections are collected with the EM grid prepared in Panel D. **F**, **G** Cryogenic SMLM. **F** Channel B (red channel) of the cryogenic fluorescence microscope is calibrated by image transformation to match Channel A (green channel) with multicolour fluorescent microspheres. **G** When the sample is illuminated by laser beams, photon emission events of single molecules are captured as a video (upper), from which a superresolution fluorescence micrograph can be reconstructed (green, lower left and lower right). Dark red fluorescent microspheres are also captured in diffraction-limited wide-field mode (red, lower right). **H** Cryo-ET. Tomographic tilt series are acquired at fluorescence-positive locations. Electron tomograms are generated from the tilt series. **I** Image registration. A fluorescence micrograph (dark red fluorescent microspheres and green fluorescence signals on a dark background), a low-magnification electron micrograph (darker grey) and a projection of a tomogram (brighter grey) are aligned to each other. See Fig. 2 for details about the image registration. Scale bars: 2 μm for Panels F and G

**Figure 2:**
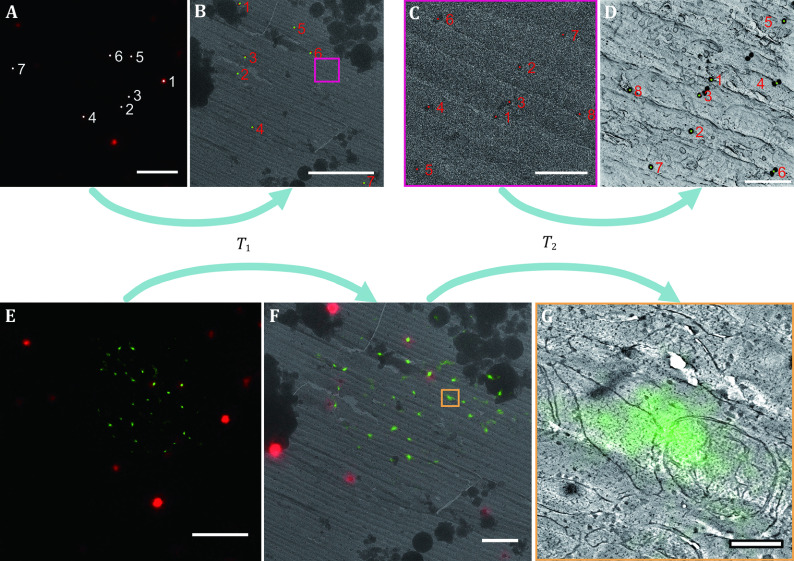
Workflow for image registration. **A**, **B** Establishment of *T*_1_ transformation that aligns the fluorescence micrograph to the low-magnification electron micrograph. Fluorescent microspheres are localised on the fluorescence micrograph (**A**, white points) and the low-magnification electron micrograph (**B**, red points, mostly covered by green points). A point pair corresponding to the same microsphere is labelled with the same number. Based on the coordinates of these point pairs, an image transformation *T*_1_ is established. Green points on Panel B mark the positions of the white points on Panel A after the *T*_1_ transformation. **C**, **D** Establishment of *T*_2_ transformation that aligns the low-magnification electron micrograph to the tomogram. The boxed area of Panel B is enlarged to be shown as Panel C. The 50 nm gold nanoparticles are localised on the low-magnification electron micrograph (**C**, red points) and the minimum intensity projection of the tomogram (**D**, red points, mostly covered by green points). A point pair corresponding to the same gold nanoparticle is labelled with the same number. An image transformation *T*_2_ is similarly established. Green points on Panel D mark the positions of the red points on Panel C after the *T*_2_ transformation. **E**–**F** Alignment of the fluorescence micrograph to the low-magnification electron micrograph. *T*_1_ is applied to the superresolution fluorescence micrograph (**E**, to better demonstrate the process, the diffraction-limited red channel is included) to obtain a transformed image that aligns with the low-magnification electron micrograph (**F**, to better demonstrate the process, the low-magnification electron micrograph is also shown as a reference). **F**–**G** Alignment of the fluorescence micrograph to a tomogram slice. *T*_2_ is applied to the transformed image (**F**), and the twice-transformed superresolution fluorescence image is superimposed on a tomogram slice to obtain Panel G. The tomogram corresponds to the boxed area in Panel F. Scale bars: 5 µm for Panels A, B and E; 500 nm for Panels C and D; 2 µm for Panel F; 200 nm for Panel G

**Figure 3:**
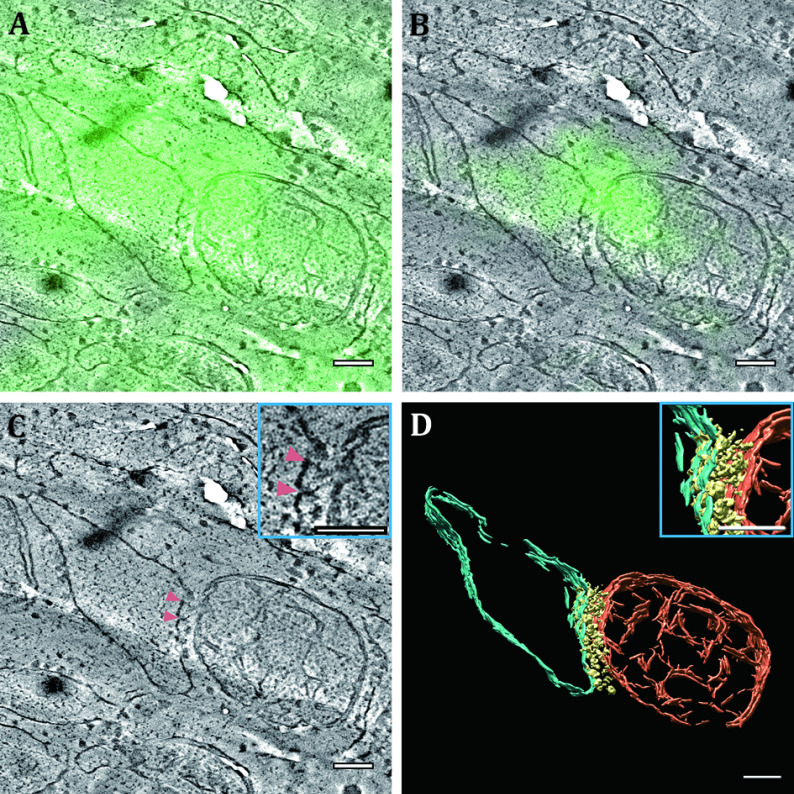
Fluorescence-annotated tomographic visualisation of an ER–mitochondrial membrane contact site. **A** When a diffraction-limited wide-field fluorescence micrograph is aligned to and superimposed on a tomogram slice, the blurred fluorescence pattern hardly presents any information. **B** When a superresolution fluorescence micrograph obtained with SMLM is aligned to and superimposed on the tomogram slice, the fluorescence marks an ER–mitochondrial membrane contact site. **C** In the position where fluorescence exists, the electron density (pink arrowheads) can be observed spanning the cleft of the membranes of the two organelles. **D** 3D segmentation of the ER membrane (cyan), the mitochondrial membranes (orange) and the density (yellow) between the ER membrane and the outer mitochondrial membrane. The insets in Panels C and D correspond to the same region. Watch Video S1 for an animated 3D presentation of Panels C and D. Scale bars: 100 nm

The complete csCLEM scheme (Fig. 1) includes four stages: sample preparation, cryogenic SMLM, cryo-ET and image registration (Fig. 2). In the end, electron tomograms annotated by superresolution fluorescence micrographs are expected to be obtained (Fig. 3).

### Sample preparation

Gold nanoparticles and fluorescent microspheres are applied over EM grids as fiducial markers for image registration and tilt series alignment (Fig. 1D). Cells with ER-mitochondrial membrane contact sites labelled by green fluorescence are cultured, collected and vitrified by high-pressure freezing (Fig. 1A–1C). The vitrified cells are sectioned with a cryo-ultramicrotome, and the cryosections are deposited onto the previously prepared EM grids (Fig. 1E).

### Cryogenic SMLM

The two fluorescence channels of the cryogenic fluorescence microscope, each for capturing the fluorescence signals of the fluorescent protein (Channel A) and fluorescent microspheres (Channel B), are calibrated with multicolour fluorescent microspheres (Fig. 1F). The EM grids holding the cryosections are loaded into the liquid nitrogen-cooled fluorescence imaging system and screened for regions of interest (ROIs). These ROIs are illuminated by an excitation laser beam and repetitive pulses of an activation laser beam, and the photon emission processes in Channel A are recorded as videos by the camera (Fig. 1G). Superresolution fluorescence micrographs are reconstructed from these videos (Fig. 1G). The fluorescence of the fluorescent microspheres in Channel B is registered to the micrographs based on the previously obtained calibration parameters (Fig. 1G).

### Cryo-ET

The samples are transferred to a cryogenic transmission electron microscope. Low-magnification electron micrographs are recorded at the squares where fluorescence imaging was performed to produce montage maps. By comparing the fluorescence patterns of the microspheres and their electron density in the EM maps, the fluorescence signals in the superresolution micrographs can be mapped onto the EM maps. Tomographic tilt series are acquired at these fluorescence-positive locations (Fig. 1H). Electron tomograms are reconstructed from the tilt series (Fig. 1H).

### Image registration

The fluorescence micrographs are aligned to the low-magnification electron micrographs according to the spatial patterns of the fluorescent microspheres (Fig. 2A and 2B). The low-magnification electron micrographs are aligned to the tomographic slices according to the spatial patterns of the gold nanoparticles (Fig. 2C and 2D). Finally, the fluorescence micrographs are transformed and superimposed onto the tomographic slices (Fig. 2E–2G).

## MATERIALS, INSTRUMENTATION AND SOFTWARE

### Cell line

The cell line used as an example in this article was described by Yang *et al*. (2018). In brief, cDNAs encoding one part of split GFP (spGFP1–10), a V5 tag and an ER anchoring sequence (residues 228–259 of protein UBE2J2) were cloned into plx304 plasmids. cDNAs encoding a mitochondrial localisation sequence (residues 1–59 of protein TOMM70), two copies of another part of split GFP (spGFP11), and the linker sequence GSGSNGSSGSGGSGGGGSGGSRGGSGGGGSGG were inserted into the pLVX-IRES-puro vector. A stable U2OS cell line was generated by lentiviral infection and subsequent selection. When the distance between the ER and a mitochondrion is small enough, the two parts of split GFP expressed in the cell will refold and emit green fluorescence.

### Reagents

• 10 nm BSA gold tracer (Electron Microscopy Sciences, cat. no. 25486)

• 50 nm spherical gold nanoparticles (Nanopartz, part no. AC11-50-NPC-DIH-100-1)

• Double distilled water

• 0.2 μm dark red fluorescent microspheres (ThermoFisher, cat. no. F8807)

• DMEM (ThermoFisher, cat. no. C11995500BT)

• Fetal bovine serum (ThermoFisher, cat. no. 10091148)

• Penicillin-streptomycin (ThermoFisher, cat. no. 15140122)

• Trypsin-EDTA (ThermoFisher, cat. no. 25300054)

• Dextran (Merck, product no. 31389)

• 0.2 µm TetraSpeck microspheres (ThermoFisher, cat. no. T7280)

### Equipment

• Vortex mixer (SCILOGEX, MX-S)

• Ultrasonic cleaner (Kunshan Shumei, KQ3200DE)

• Modified 200 mesh copper EM finder grids described in Xu’s doctoral dissertation (2019)

• Pipettes and pipette tips

• Filter paper (NEWSTAR, ⌀ 12.5 cm qualitative filter paper)

• EM grid boxes

• Super clean bench (Yataikelong, KLCZ-1220A)

• Cell incubator (ThermoFisher, Forma Series II Water Jacket CO_2_ Incubator)

• Cell culture dishes (ThermoFisher, Nunc EasYDish Dishes)

• Centrifuge (ZONKIA, SC-2554)

• Centrifuge tubes

• High pressure freezer and accessories (Leica, EM HPM100)

• Liquid nitrogen and containers

• Cryo-ultramicrotome (Leica EM UC7/FC7) with an electrostatic charger (Leica EM CRION)

• Manipulator (DiATOME)

• 45° diamond knife (DiATOME, trim 45) and 25° diamond knife (DiATOME, cryo 25°)

• Cryo-EM grid boxes

• Cryogenic fluorescence imaging system and accessories described by Xu (2019) and Xu *et al*. (2018)

• CUDA supported personal computer

• 300 kV cryogenic transmission electron microscope and accessories (FEI, Titan Krios)

### Software

• Andor Solis camera control software platform (for the cryogenic fluorescence imaging system) (https://andor.oxinst.com/products/solis-software/)

• Custom-designed programs for stage movement control, laser manipulation and temperature monitoring (for the cryogenic fluorescence imaging system)

• ImageJ (https://imagej.nih.gov/ij/) with the MultiStackReg plugin (http://bradbusse.net/sciencedownloads.html)

• CUDA Toolkit (https://developer.nvidia.com/cuda-toolkit)

• MATLAB (https://www.mathworks.com/products/matlab.html)

• Custom-developed MATLAB program package for SMLM reconstruction (Liu *et al*. 2015; Xu *et al*. 2018; Zhang *et al*. 2013)

• Custom-developed MATLAB program package for image registration (https://github.com/ZergTBY/Bioimage/tree/master/POIregistration)

• SerialEM (https://bio3d.colorado.edu/SerialEM/)

• MotionCor2 (https://emcore.ucsf.edu/ucsf-software)

• IMOD (https://bio3d.colorado.edu/imod/)

• EMAN2 (https://blake.bcm.edu/emanwiki/EMAN2)

• UCSF Chimera (https://www.cgl.ucsf.edu/chimera/)

## PROCEDURE

### Stage 1: Sample preparation [TIMING ~ 1 week]

#### Step 1.1: Application of fiducial markers over the EM finder grids [TIMING 1–2 h]

Vortex 10 nm BSA gold tracer/50 nm spherical gold nanoparticles/double distilled water (1:2:3, *v*/*v*/*v*) for 30 s. Ultrasonicate the mixture for at least 15 min to disperse the gold particles. Add 0.2 μm dark red fluorescent microspheres to the mixture (1:80, *v*/*v*) and vortex it for 30 s to create a suspension. Gently apply 5 μL of the suspension to the film of each EM finder grid with a pipette. After waiting for 1 min, remove any excess suspension by blotting the grid from the side with a piece of filter paper. After the grids are air-dried, store them in an EM gird box.

**[CRITICAL STEP]** It is recommended that the treated side of all of the grids in the box face the same direction. Remember this orientation. In the following steps, cryosections will be placed on the treated side.

**[NOTE]** This step does not have to precede Steps 1.2 & 1.3, so long as it is completed before Step 1.4.

**[? TROUBLESHOOTING]**

#### Step 1.2: Culture of fluorescence-labelled cells [TIMING 3–5 d]

Culture U2OS cells with stable ER-mitochondrial contact reporter expression (Yang *et al*. 2018) in DMEM supplemented with 10% fetal bovine serum and 1% penicillin-streptomycin. Incubate the cell culture at 37 °C and 5% CO_2_ level in a humidified incubator.

**[NOTE]** Other cells with different fluorescent labels may fit this protocol if the fluorophore used is photoactivable at cryogenic temperature.

#### Step 1.3: High-pressure freezing [TIMING 2-4 h]

Harvest the cells with trypsin-EDTA. Centrifuge the cell suspension at 300 *g* for 5 min. Resuspend the cells in 40% *w*/*v* dextran. Load the suspension into capillary copper tubes and vitrify the sample with a high-pressure freezer. Collect and store the copper tubes containing the cell sample in liquid nitrogen.

**[CRITICAL STEP]** To ensure successful vitrification, the freezing pressure and the cooling rate during high-pressure freezing should be checked. For the Leica EM HPM100, the freezing pressure should be ~2,100 bar, and the cooling rate should range between 12,000 K/s and 25,000 K/s.

#### Step 1.4: Cryo-ultramicrotomy [TIMING 1–2 d]

Cool the cryochamber of a cryo-ultramicrotome to 123 K (–150 °C). Mount a copper tube containing the vitrified cells in the holder of the cryo-ultramicrotome. Trim the sides of the copper tube with a 45° diamond knife to expose the vitrified cell sample inside. Section the sample into cryosections with a nominal thickness of 120 nm using a 25° diamond knife and obtain the ribbon of sections with the manipulator. Attach the ribbon to an EM grid prepared in Step 1.1 using the charge mode of the electrostatic charger. Repeat the sectioning-collecting cycle until enough cryosections on EM grids are produced. Place the grids holding the cryosections in cryo-EM grid boxes. Transfer and store them in liquid nitrogen.

**[CRITICAL STEP]** Cryosections should be attached to the side of the grids over which the fluorescent microspheres are applied; otherwise, the fluorescent microspheres will be out of focus during the fluorescence imaging.

### Stage 2: Cryogenic SMLM [TIMING ~ 1 week: ~ 4 d for Steps 2.1–2.4, ~ 3 d for Steps 2.5 and 2.6]

#### Step 2.1: Initialisation of the cryogenic fluorescence imaging system

Cool the cryochamber by steadily pumping liquid nitrogen in with the NORHOF micro dosing LN2 system. Stabilise the temperatures of the objective and the sample stage at 300 K (27 °C) and 93 K (–180 °C), respectively, with the temperature controller.

**[CRITICAL STEP]** The temperatures must be stabilised to ensure the mechanical stability of the imaging system. Ideally, temperature fluctuations should not exceed approximately 0.03 K within approximately 30 min. Otherwise, the mechanical drift induced by temperature fluctuations becomes so obvious that SMLM cannot be performed.

**[? TROUBLESHOOTING]**

#### Step 2.2: Calibration of fluorescence channels

Dilute TetraSpeck microspheres (0.2 µm) with double distilled water (1:50, *v*/*v*). Vortex the suspension for 30 s. Add 5 μL of the suspension to a fresh EM grid and air-dry it. Load this grid into the sample holder and then place the holder on the sample stage. Initialise the Andor Solis software platform and allow the camera to cool to 203 K (–70 °C). Illuminate a field of view with 488 nm and 561 nm laser beams such that the TetraSpeck microspheres are visible in both channels of the camera. Capture an image that contains both channels. Align the subimage for Channel B (red fluorescence) to the subimage for Channel A (green fluorescence and bright field) with the MultiStackReg plugin for ImageJ selecting “Affine” transformation mode. Save the transformation file that records the parameters for the transformation. In subsequent imaging steps, correct all subimages for Channel B with the MultiStackReg plugin using the saved parameters.

**[NOTE]** The EM grid with TetraSpeck microspheres used in this step can be retrieved and stored at room temperature for reuse.

#### Step 2.3: Wide-field cryogenic fluorescence imaging

Load a sample prepared in Step 1.4 into the sample holder in liquid nitrogen and place the sample holder on the sample stage. Screen the ribbon of sections under 488 nm laser illumination in search of green fluorescence-positive regions. Once an ROI is found, turn on the wide-field LED illumination to show its position relative to an alphanumeric landmark. Record this position. Illuminate the region with a 488 nm laser beam and capture a fluorescence micrograph. Align the subimage for Channel B, which displays 0.2 μm dark red fluorescent microspheres, to the subimage for Channel A, which displays green fluorescence (see Step 2.2).

**[CRITICAL STEP]** An ROI should contain sufficient 0.2 μm dark red fluorescent microspheres in focus as landmarks for FM/EM image registration. It should not be too close to the grid bars to allow subsequent tilt series acquisition in cryo-ET.

#### Step 2.4: Acquisition of SMLM data

Illuminate the ROI with a 488 nm laser beam. Increase the power density to approximately 1.5–2.0 kW/cm^2^ to bleach the green fluorescence until it is reduced to the single-molecule level. Set the EMCCD to “Kinetic” acquisition mode, “Exposure Time” to 0.05 s and “Electron Multiplier Gain Level” to 300. Start the acquisition of a video. Apply repetitive pulses of a 405 nm laser beam to activate the fluorophores during the imaging process, adjusting the frequency of pulsing and the intensity of the 405 nm laser beam to ensure that the fluorescence-emitting fluorophores are kept at a moderate density. Terminate the acquisition process when emission events become sparse and rare. Move to another ROI and repeat the wide-field imaging described in Step 2.3 and the video acquisition described in this step until all of the ROIs have been imaged. When all of the sample-holding EM grids have been imaged, transfer them back into cryogenic EM grid boxes and store them in liquid nitrogen.

**[CRITICAL STEP]** A higher power density of the 488 nm laser beam accelerates the imaging process but may devitrify the sample. The highest tolerable power density depends on the sample and the properties of the grid film. It is recommended that some trials should be conducted outside the ROIs before acquiring SMLM data.

**[? TROUBLESHOOTING]**

#### Step 2.5: File preparation for reconstruction of superresolution fluorescence micrographs

Open an acquired video as an image stack in ImageJ. Duplicate the subarea for Channel A, add a blank (dark) frame to the start of the image stack and save the image stack to a new TIFF file named [FILENAME].tif. Crop a subarea in Channel B that contains a single 0.2 μm dark red fluorescent microsphere, open the wide-field fluorescence micrograph captured in Step 2.3, crop the corresponding subarea, add it to the start of the cropped image stack and save the image stack to a new TIFF file named [FILENAME]_beads.tif. Prepare more files for the remaining ROIs.

**[CRITICAL STEP]** The first saved file is for the reconstruction of a superresolution micrograph, and the second saved file is for drift correction in the reconstruction process. It is possible to reconstruct a drift-corrected superresolution micrograph without the added initial frames of the image stacks. However, in this case, the reconstructed superresolution micrograph registers with the first frame of the raw image stack rather than the wide-field fluorescence micrograph. In the following steps, FM/EM image registration is based on the spatial coordinates on the wide-field micrograph, so it is necessary to align the reconstructed superresolution micrograph to the wide-field micrograph by introducing an initial frame in drift correction.

#### Step 2.6: Reconstruction of superresolution fluorescence micrographs

Run the main2.m script in the MATLAB program package for SMLM image reconstruction. In the GUI, open the [FILENAME].tif file and set an appropriate value for each parameter (see supplementary Table S1 for suggested values). After automatically finding particles in all of the frames, click to execute the linking, fitting and reconstruction steps. Merge the reconstructed superresolution micrograph with the calibrated subimage for Channel B obtained in Step 2.3 after scaling the latter to the same scale. Process the remaining data for the other ROIs likewise.

**[? TROUBLESHOOTING]**

### Stage 3: Cryo-ET [TIMING variable: 24 h for Steps 3.1 and 3.2, variable time for Step 3.3]

#### Step 3.1: Acquisition of low-magnification EM maps

Load the sample grids into a Titan Krios 300 kV cryogenic transmission electron microscope. Find a grid square containing an ROI with the help of the alphanumeric landmarks. Add stage positions in a SerialEM navigator to draw a polygon that specifies a region for map acquisition. Acquire a montage map in low dose mode at 3600× magnification.

#### Step 3.2: Acquisition of tomographic tilt series

Visually compare the merged fluorescence micrograph obtained in Step 2.6 with the EM map obtained in Step 3.1. By inspecting the relative positions of the 0.2 μm dark red fluorescent microspheres on the fluorescence micrograph and the EM map, establish a spatial correlation between the two images. Find the corresponding positions of the green fluorescence spots on the EM map and add points to those positions in the SerialEM navigator. Acquire tilt series with SerialEM at these points with the following settings: magnification = 26,000, pixel size = 5.424 Å, target defocus = 0 μm, starting tilt angle = –51°, increment = 3°, ending tilt angle = 51°, total dose ≈ 100 e^−^/Å^2^, number of subframes = 20, slit width of the energy filter = 20 eV and with Volta phase plate on. Then, go to another ROI, acquire a map and repeat this step.

**[NOTE]** The settings may vary according to need.

#### Step 3.3: Reconstruction and visualisation of electron tomograms

Refine the raw images by performing motion correction with MotionCor2 (Zheng *et al*. 2017). Group the corrected images by tilt series and make image stacks with the “newstack” program in IMOD package. Generate tomograms with IMOD (Mastronarde and Held 2017). If necessary, segment the tomograms with EMAN2 (Chen *et al*. 2017), and visualise and refine the segmentations with UCSF Chimera (Goddard *et al*. 2007; Pettersen *et al*. 2004).

**[NOTE]** A thorough description of all the details of software manipulation in this step is beyond the scope of this article. Detailed instructions for each of these program packages can be found at the following addresses.

MotionCor2: go to https://emcore.ucsf.edu/ucsf-software, download the software package and see the manual included in the ZIP file.

IMOD: see the tomography guide at https://bio3d.colorado.edu/imod/doc/tomoguide.html.

EMAN2: see the tutorial for tomogram segmentation at https://blake.bcm.edu/emanwiki/EMAN2/Programs/tomoseg.

UCSF Chimera: see the guide to volume data display at https://www.cgl.ucsf.edu/chimera/data/tutorials/volumetour/volumetour.html.

### Stage 4: Image registration [TIMING 2–4 d]

See Fig. 2 for a graphic explanation of the following steps.

#### Step 4.1: Registration between FM images and low-magnification electron micrographs [TIMING 1–2 d]

Run the FLBeadReg_103.m script in the MATLAB program package for image registration, selecting the “Projective” transformation mode. Select a Channel B fluorescence image displaying 0.2 μm dark red fluorescent microspheres obtained in Step 2.3 as the image to be transformed. Select a corresponding low-magnification electron micrograph, which is a single piece of an EM map obtained in Step 3.1, as the reference image. Draw a rectangle on the fluorescence image that contains a spot of fluorescence emitted by a single fluorescent microsphere, and the script will automatically localise the centre of the fluorescent microsphere. Click on the electron micrograph to pinpoint the centre of the corresponding microsphere. Repeat this process until all or enough fluorescent microspheres are localised. Proceed to generate a Transformation.mat file that automatically records the parameters for the image transformation. Run the FLBeadReg_103.m script again. Select the corresponding superresolution fluorescence micrograph as the image to be transformed. Select the low-magnification electron micrograph as the reference image. Load the Transformation.mat file, and the script will automatically transform the superresolution fluorescence micrograph to align it to the electron micrograph. Process the remaining data likewise.

**[CRITICAL STEP]** If the wide-field Channel B fluorescence image and the superresolution fluorescence image have inconsistent scales (which is the usual case), first interpolate the former to match the scale before any manipulation.

#### Step 4.2: Registration between low-magnification and high-magnification electron micrographs [TIMING 1–2 d]

Open a tomogram produced in Step 3.3 in ImageJ and make a minimum intensity projection to show the projective positions of the 50-nm gold nanoparticles. Run the POIRegistration_103.m script in the MATLAB program package for image registration, selecting the “Projective” transformation mode. Select the low-magnification electron micrograph as the image to be transformed. Select the minimum intensity projection of the tomogram as the reference image. Manually select the centres of the 50-nm gold nanoparticles on both images as control point pairs for image transformation. Proceed to generate a Transformation.mat file that automatically records the parameters for the image transformation. Process the remaining data likewise.

#### Step 4.3: Generation of superresolution fluorescence-annotated tomographic slices [TIMING ~ 2 h]

Run the POIRegistration_103.m script again. Select a transformed superresolution fluorescence micrograph that was aligned to a corresponding low-magnification electron micrograph from Step 4.1 as the image to be transformed. Select a tomogram slice as the reference image. Load the Transformation.mat file obtained in Step 4.2 and the script will automatically retransform the transformed superresolution fluorescence micrograph to align it to the tomogram slice and merge the two. Process the remaining data likewise.

**[TIMING]**

Stage 1, sample preparation: ~ 1 week

Step 1.1, application of fiducial markers over EM finder grids: 1–2 h

Step 1.2, culture of fluorescence-labelled cells: 3–5 d

Step 1.3, high-pressure freezing: 2–4 h

Step 1.4, cryo-ultramicrotomy: 1–2 d

Stage 2, cryogenic SMLM: ~ 1 week

Steps 2.1–2.4, imaging samples with the cryogenic fluorescence imaging system: ~ 4 d

Steps 2.5 and 2.6, image reconstruction: ~ 3 d

Stage 3, cryo-ET: variable time

Steps 3.1 and 3.2, acquisition of EM data: 24 h

Step 3.3, reconstruction and visualisation of electron tomograms: variable time

Stage 4, image registration: 2–4 d

Step 4.1, registration between FM images and low-magnification electron micrographs: 1–2 d

Step 4.2, registration between low-magnification and high-magnification electron micrographs: 1–2 d

Step 4.3, generation of superresolution fluorescence-annotated tomographic slices: ~ 2 h

**[? TROUBLESHOOTING]**

Troubleshooting advice can be found in Table 1.

**Table 1:** Troubleshooting

Step	Problem	Possible cause	Solution
1.1	Fibrous contaminants on grid films	If the edge of the filter paper is not smooth and clean, it may spread fibrous contaminants to the suspension when touching it	Take a new piece of filter paper, gently fold it and use the folding edge to blot the grid
2.1	The temperature of the sample stage cannot be stabilised at 93 K (–180 °C)	The liquid nitrogen inflow is not stable or not sufficiently strong and the temperature of the cryo chamber is consequently not stabilised	Adjust the pressure of the liquid nitrogen pump to a moderate level
2.4	The sample falls out of focus during video acquisition	The temperature of the sample stage is not stabilised so a noticeable mechanical drift occurs	Stabilise the temperature (see above)
		Unspecified environmental disturbances destabilise the stage	Terminate the acquisition, refocus by adjusting stage Z and restart the acquisition
2.6	Error report	The entire folder of the program package is not added to the path	Add the entire folder, including all of the subfolders, to the path before running the main2.m script
		CUDA is not properly installed	Reinstall the CUDA Toolkit

## ANTICIPATED RESULTS

When Stage 2 is completed, SMLM-based superresolution fluorescence micrographs should be obtained. A representative image is shown in Fig. 2E, with which the diffraction-limited red channel is merged displaying dark red fluorescent microspheres. For the demonstrated data, the mean localisation error in SMLM is about 17 nm.

By going through the steps in Stage 3, low-magnification electron micrographs (see Fig. 2B and 2C as an example) and high-magnification electron tomograms (see Fig. 3C and Video S1 as an example) should be acquired. If some of the tomograms are segmented in Step 3.3, 3D models of structures of interest are expected to be obtained (see Fig. 3D and Video S1 as an example).

After Stage 4 (depicted in Fig. 2), the superresolution fluorescence micrographs should be aligned to and merged with the tomogram slices (see Fig. 3B as an example). To estimate the registration errors, the displacements of the control point pairs in the two transformations are measured and analysed. For the demonstrated data, the root mean square error of the first transformation in Step 4.1 is approximately 57 nm and that of the second transformation in Step 4.2 is approximately 4.5 nm. These lateral localisation and registration errors are small enough for SMLM to pinpoint the lateral position of a target in a tomogram at the suborganelle level. In Fig. 3B, the fluorescence marks an ER–mitochondrial membrane contact site. In the corresponding position in Fig. 3C, electron density can be seen spanning the cleft of the two membranes.

## Abbreviations

**Table:** 

CLEM	Correlative light and electron microscopy
EM	Electron microscopy
FM	Fluorescence microscopy
SMLM	Single-molecule localisation microscopy
ET	Electron tomography
csCLEM	Cryogenic superresolution correlative light and electron microscopy
ER	Endoplasmic reticulum
ROI	Region of interest

## Conflict of interest

Buyun Tian, Maoge Zhou, Fengping Feng, Xiaojun Xu, Pei Wang, Huiqin Luan, Wei Ji, Yanhong Xue and Tao Xu declare that they have no conflict of interest.

## References

[bAsano2015] (2015). *In situ* cryo-electron tomography: a post-reductionist approach to structural biology. J Mol Biol.

[bBetzig2006] (2006). Imaging intracellular fluorescent proteins at nanometer resolution. Science.

[bBriegel2010] (2010). Correlated light and electron cryo-microscopy. Methods Enzymol.

[bChang2014] (2014). Correlated cryogenic photoactivated localization microscopy and cryo-electron tomography. Nat Methods.

[bChen2017] (2017). Convolutional neural networks for automated annotation of cellular cryo-electron tomograms. Nat Methods.

[bDahlberg2021] (2021). Cryogenic super-resolution fluorescence and electron microscopy correlated at the nanoscale. Annu Rev Phys Chem.

[bDahlberg2020] (2020). Cryogenic single-molecule fluorescence annotations for electron tomography reveal in situ organization of key proteins in *Caulobacter*. Proc Natl Acad Sci USA.

[bde2015] (2015). Correlated light and electron microscopy: ultrastructure lights up!. Nat Methods.

[bDeRosier2021] (2021). Where in the cell is my protein. Q Rev Biophys.

[bFeynman1960] (1960). There’s plenty of room at the bottom. Eng Sci.

[bFu2020] (2020). mEosEM withstands osmium staining and Epon embedding for super-resolution CLEM. Nat Methods.

[bGoddard2007] (2007). Visualizing density maps with UCSF Chimera. J Struct Biol.

[bHess2006] (2006). Ultra-high resolution imaging by fluorescence photoactivation localization microscopy. Biophys J.

[bHoffman2020] (2020). Correlative three-dimensional super-resolution and block-face electron microscopy of whole vitreously frozen cells. Science.

[bPettersen2004] (2004). UCSF chimera — A visualization system for exploratory research and analysis. J Comput Chem.

[bKukulski2012] (2012). Precise, correlated fluorescence microscopy and electron tomography of Lowicryl sections using fluorescent fiducial markers. Methods Cell Biol.

[bLi2018] (2018). The application of CorrSight^TM^ in correlative light and electron microscopy of vitrified biological specimens. Biophys Rep.

[bLiu2015] (2015). Three-dimensional super-resolution protein localization correlated with vitrified cellular context. Sci Rep.

[bLucic2005] (2005). Structural studies by electron tomography: From cells to molecules. Annu Rev Biochem.

[bMastronarde2017] (2017). Automated tilt series alignment and tomographic reconstruction in IMOD. J Struct Biol.

[bMilne2013] (2013). Cryo-electron microscopy — A primer for the non-microscopist. FEBS J.

[bOrlova2011] (2011). Structural analysis of macromolecular assemblies by electron microscopy. Chem Rev.

[bPaezSegala2015] (2015). Fixation-resistant photoactivatable fluorescent proteins for CLEM. Nat Methods.

[bRust2006] (2006). Sub-diffraction-limit imaging by stochastic optical reconstruction microscopy (STORM). Nat Methods.

[bScher2021] (2021). 50 Shades of CLEM: how to choose the right approach for you. Methods Cell Biol.

[bSchwartz2007] (2007). Cryo-fluorescence microscopy facilitates correlations between light and cryo-electron microscopy and reduces the rate of photobleaching. J Microsc.

[bTian2021] (2021). Cryogenic superresolution correlative light and electron microscopy on the frontier of subcellular imaging. Biophys Rev.

[bTuijtel2019] (2019). Correlative cryo super-resolution light and electron microscopy on mammalian cells using fluorescent proteins. Sci Rep.

[bWang2017] (2017). Using integrated correlative cryo-light and electron microscopy to directly observe syntaphilin-immobilized neuronal mitochondria *in situ*. Biophys Rep.

[bWatanabe2011] (2011). Protein localization in electron micrographs using fluorescence nanoscopy. Nat Methods.

[bXu2019] Xu XJ (2019) The construction of the ultra-stable super-resolution fluorescence cryo-microscopy and the development of the support film for correlative light and electron cryo-microscopy. Dissertation, Huazhong University of Science and Technology

[bXu2018] (2018). Ultra-stable super-resolution fluorescence cryo-microscopy for correlative light and electron cryo-microscopy. Sci China Life Sci.

[bYang2018] (2018). A novel fluorescent reporter detects plastic remodeling of mitochondria–ER contact sites. J Cell Sci.

[bZhang2013] (2013). . Ultrafast, accurate, and robust localization of anisotropic dipoles. Protein Cell.

[bZheng2017] (2017). MotionCor2: anisotropic correction of beam-induced motion for improved cryo-electron microscopy. Nat Methods.

